# Nephrological, Pulmonary, and Dermatological Complications in the Context of MAFLD/NAFLD: A Narrative Review

**DOI:** 10.3390/metabo15040272

**Published:** 2025-04-14

**Authors:** Vlad Pădureanu, Dalia Dop, Lucrețiu Radu, Dumitru Rădulescu, Rodica Pădureanu, Denisa Floriana Vasilica Pîrșcoveanu, Daniel Cosmin Caragea

**Affiliations:** 1 Department of Internal Medicine, University of Medicine and Pharmacy Craiova, 200349 Craiova, Romania; vlad.padureanu@umfcv.ro; 2 Department of Pediatrics, University of Medicine and Pharmacy Craiova, 200349 Craiova, Romania; dalia.dop@umfcv.ro; 3 Department of Hygiene, University of Medicine and Pharmacy Craiova, 200349 Craiova, Romania; lucretiu.radu@umfcv.ro; 4 Department of Surgery, University of Medicine and Pharmacy Craiova, 200349 Craiova, Romania; 5 Department of Neurology, University of Medicine and Pharmacy Craiova, 200349 Craiova, Romania; denisa.pirscoveanu@umfcv.ro; 6 Department of Nephrology, University of Medicine and Pharmacy Craiova, 200349 Craiova, Romania; daniel.caragea@umfcv.ro

**Keywords:** non-alcoholic fatty liver disease (NAFLD), metabolic-associated fatty liver disease (MAFLD), chronic kidney disease, obstructive sleep apnea, psoriasis

## Abstract

**Background**: The most common cause of chronic liver disease is now known to be non-alcoholic fatty liver disease (NAFLD), recently redefined as metabolic-associated fatty liver disease (MAFLD). This review aims to synthesize current evidence on the pathophysiology and clinical implications of nephrological, pulmonary, and dermatological manifestations among NAFLD/MAFLD patients. In order to find safe and efficient treatments, NAFLD/MAFLD has emerged as a primary concern for hepatologists worldwide. **Methods**: We conducted a comprehensive review of the literature from major databases, focusing on studies that evaluated the extrahepatic manifestations of NAFLD/MAFLD. Emphasis was placed on identifying pathophysiological mechanisms and assessing their clinical impact on renal, pulmonary, and dermatological systems. **Results**: Recent developments in the management of chronic viral hepatitis have lowered the mortality rate associated with chronic liver disease. However, the prevalence of NAFLD/MAFLD continues to rise, making chronic liver disease a significant health concern for the future. An increasing percentage of patients on liver transplant waiting lists now have cirrhosis and hepatocellular carcinoma due to non-alcoholic liver disease. Furthermore, the incidence and prevalence of chronic kidney disease have surged, linking NAFLD/MAFLD to higher morbidity, mortality, and healthcare costs. **Conclusions**: NAFLD/MAFLD is underdiagnosed and underappreciated, yet its incidence is rapidly increasing, raising concerns about a potential global epidemic. Given its multisystemic impact—extending to renal, pulmonary, and dermatological complications—it is crucial to develop interdisciplinary strategies for early detection and effective management of the disease.

## 1. Introduction

Metabolic-associated fatty liver disease (MAFLD), formerly known as nonalcoholic fatty liver disease (NAFLD), is a spectrum of liver conditions that range from simple steatosis to non-alcoholic steatohepatitis (NASH), potentially progressing to cirrhosis and hepatocellular carcinoma. MAFLD/NAFLD is strongly associated with components of metabolic syndrome (obesity, insulin resistance, hypertension, dyslipidemia) and has emerged as one of the most prevalent chronic liver diseases worldwide [1,2,3]. Over the last decade, accumulating evidence indicates that MAFLD/NAFLD is not confined to the liver alone, but has systemic repercussions [4]. Its impact on the renal, pulmonary, and skin systems has gained increasing attention, largely due to the shared pathophysiological mechanisms of insulin resistance, chronic inflammation, and ectopic fat deposition [5]. Chronic kidney disease (CKD) and NAFLD are global public health issues due to their rising incidence, unfavorable prognoses, and high medical costs [6,7]. Moderate to advanced stages of chronic kidney disease (CKD) in patients with ultrasound-detected NAFLD were independently linked to higher all-cause mortality over a mean follow-up period of 19 years, according to a recent analysis of the Third National Health and Nutrition Survey database, which included approximately 11,700 American subjects [8]. Regardless of the existence or lack of established risk factors for conditions such as obesity, hypertension, type 2 diabetes mellitus (T2DM), or metabolic syndrome, several investigations conducted in the past ten years have shown the link between NAFLD and CKD [9,10]. Patients with NAFLD have high rates of chronic kidney disease (CKD), ranging from 20% to 50%, while those without the disease have rates of 5% to 25% [7,11,12]. Recognizing these extrahepatic manifestations is crucial for integrated patient care and for preventing organ-specific complications. This review briefly covers the pathophysiology and clinical implications of nephrological, pulmonary, and dermatological manifestations among NAFLD patients.

In recent years, several review articles have examined the conceptual transition from NAFLD to MAFLD and the implications of this change for understanding both hepatic and extrahepatic complications. For example, recent works have emphasized the relationships between MAFLD and cardiovascular disease, metabolic syndrome, and chronic kidney disease. Notably, reviews by Boccatonda et al. [13] and Quek et al. [14] extensively documented the cardiovascular and renal implications of MAFLD, highlighting the increased risk of global mortality and systemic complications. Additionally, a meta-analysis by Pennisi et al. [15] compared the risks associated with NAFLD and MAFLD, focusing on cardiovascular disorders, CKD, and extrahepatic cancers.

In contrast to these syntheses—which predominantly focus on cardiometabolic and renal comorbidities—the present manuscript offers an original contribution by expanding the analysis to include the pulmonary and dermatological impacts of NAFLD/MAFLD. In particular, this review explores potential connections with respiratory conditions such as obstructive sleep apnea and dermatologic diseases like psoriasis, areas that have received relatively less attention in recent literature. This integrative approach not only provides a more extensive understanding of the multisystemic nature of MAFLD but also underscores the necessity for an interdisciplinary evaluation in the management of affected patients.

## 2. Materials and Methods

### 2.1. Search Strategy and Data Sources

An extensive literature search was conducted using the PubMed, Scopus, and Web of Science databases to identify studies addressing the extrahepatic complications of metabolic-associated fatty liver disease (MAFLD), formerly known as nonalcoholic fatty liver disease (NAFLD). The search spanned publications from January 2014 through January 2025. We employed keywords such as “MAFLD”, “NAFLD”, and “non-alcoholic fatty liver disease” in combination with “chronic kidney disease”, “CKD”, “pulmonary manifestations”, “obstructive sleep apnea”, “respiratory complications”, “dermatological manifestations”, “psoriasis”, and “skin involvement.” Boolean operators and truncation symbols were used to refine the search, which initially yielded 5620 records.

### 2.2. Screening and Study Selection

**Figure 1:**
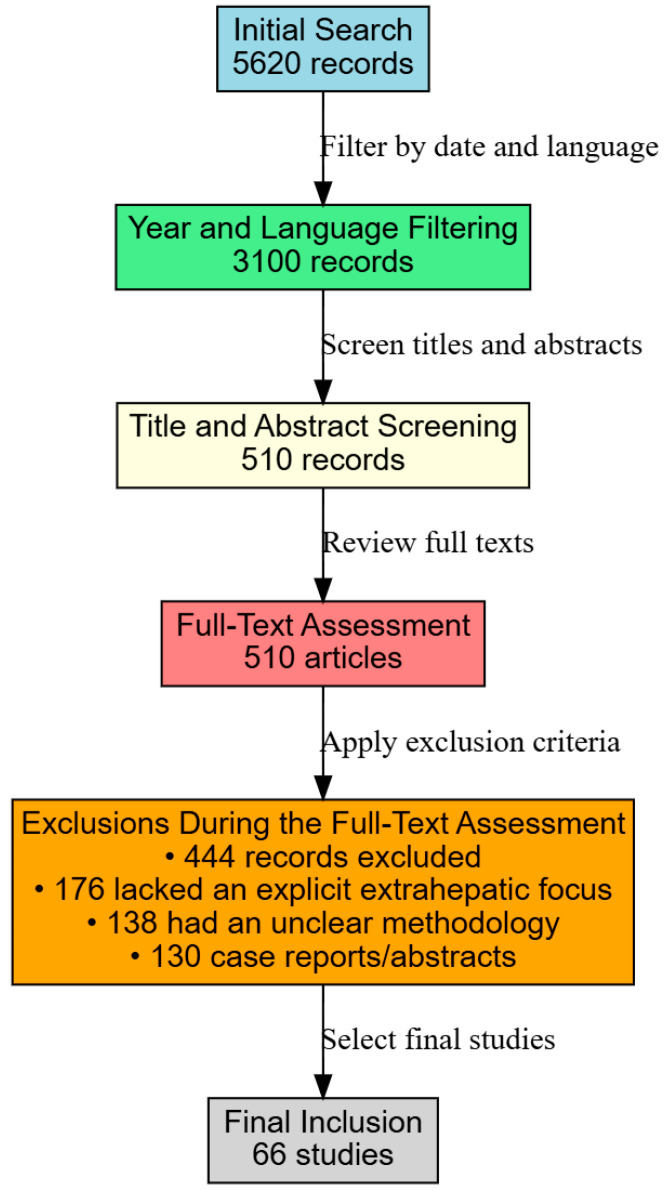
Study flowchart.

After applying filters for publication date and English language, the number of records was narrowed down to 3100 (Figure 1).

Two reviewers independently screened titles and abstracts, excluding studies focusing solely on alcohol-related liver diseases, viral hepatitis, or pediatric liver disorders. This screening reduced the pool to 510 articles deemed suitable for full-text review. A detailed evaluation of these full texts was then undertaken to confirm their relevance to our focus on renal, pulmonary, and dermatological outcomes. Articles that did not explicitly discuss extrahepatic complications, had unclear methodologies, or were limited to case reports or conference abstracts were excluded, resulting in 444 records being removed.

### 2.3. Inclusion Criteria and Final Selection

Studies were included if they addressed adult patients diagnosed with MAFLD/NAFLD, provided clear diagnostic criteria and outcome measures for extrahepatic manifestations (e.g., eGFR for renal function, polysomnography for sleep apnea, and clinical or histological assessments for dermatological conditions), and were published as original research, systematic reviews, or meta-analyses. Following this rigorous selection process, 66 studies met all inclusion criteria and were incorporated into this narrative review. Although our process did not strictly adhere to PRISMA guidelines, it was executed thoroughly to ensure the comprehensive synthesis of the current evidence regarding the nephrological, pulmonary, and dermatological complications of MAFLD/NAFLD.

## 3. Pathophysiology of MAFLD/NAFLD and Its Systemic Impact

Insulin resistance is a hallmark of metabolic syndrome and drives hepatic steatosis. Chronic low-grade inflammation and altered adipokine profiles play key roles in disease progression [4]. The same proinflammatory mediators (TNF-α, IL-6) can adversely affect the kidneys and lungs, leading to multi-organ dysfunction.

Ectopic fat accumulation in skeletal muscle, as well as the heart, pancreas, and kidneys, can promote organ-specific dysfunction [2]. Local and systemic inflammation, oxidative stress, and impaired mitochondrial function contribute to this multifaceted pathology.

Dysbiosis and increased gut permeability can promote a proinflammatory environment and influence metabolic pathways [16]. This chronic inflammation affects not just the liver but also other organs, including the kidneys.

In addition, insulin resistance and chronic inflammation can upregulate the renin–angiotensin–aldosterone system (RAAS) [17]. Aside from contributing to hypertension, RAAS dysregulation has direct deleterious effects on renal microvasculature and fluid balance, potentially impacting pulmonary function as well.

Hepatic and peripheral insulin resistance plays a central role in the pathogenesis of NAFLD/MAFLD. At the hepatic level, insulin loses its capacity to inhibit gluconeogenesis while continuing to stimulate de novo lipogenesis, thereby contributing to the accumulation of triglycerides in hepatocytes [18]. In parallel, insulin resistance in adipose tissue promotes lipolysis, releasing free fatty acids to the liver and exacerbating hepatic steatosis [19].

Lipotoxicity ensues when the liver can no longer safely store free fatty acids as triglycerides, leading to the accumulation of toxic metabolites (e.g., ceramides, diacylglycerols). These induce oxidative stress, mitochondrial dysfunction, and apoptosis, which favor the progression to NASH and fibrosis [20]. Over the long term, this stress promotes the activation of hepatic stellate cells and excessive deposition in the extracellular matrix, contributing to progressive fibrosis [21].

An imbalance in adipokines—particularly elevated levels of leptin (proinflammatory) and reduced levels of adiponectin (anti-inflammatory)—contributes to both local and systemic inflammation. Leptin activates immune cells and hepatic stellate cells, while adiponectin protects hepatocytes from oxidative stress and lipotoxicity. This imbalance reflects a profound dysfunction of adipose tissue in MAFLD [22,23].

The intestinal microbiome also influences disease progression through dysbiosis, increased production of endotoxins (e.g., lipopolysaccharides), and defective metabolism of bile and fructose. These effects lead to increased intestinal permeability and trigger a hepatic inflammatory response, fueling the transition from steatosis to NASH [24,25].

Finally, the differences between “simple steatosis” and advanced forms (NASH, cirrhosis, HCC) are fundamental in clinical practice. Simple steatosis involves merely the accumulation of fat in hepatocytes without inflammation or cellular injury, whereas NASH is characterized by inflammation, hepatocellular ballooning, and cell death followed by fibrosis. Continued progression can lead to cirrhosis and even hepatocellular carcinoma (HCC), even in the absence of overt advanced fibrosis [26,27].

To quantify the relative contribution of each pathophysiological mechanism in MAFLD/NAFLD complications, we systematically reviewed relevant studies (Table 1). For each mechanism—such as insulin resistance, chronic inflammation, adipokine imbalance, oxidative stress, and RAAS activation—we extracted reported effect sizes, significance levels, and qualitative assessments. Based on the consistency and magnitude of these findings, we assigned a score on a 1–10 scale, where a higher score indicates a stronger contribution.

**Figure 2:**
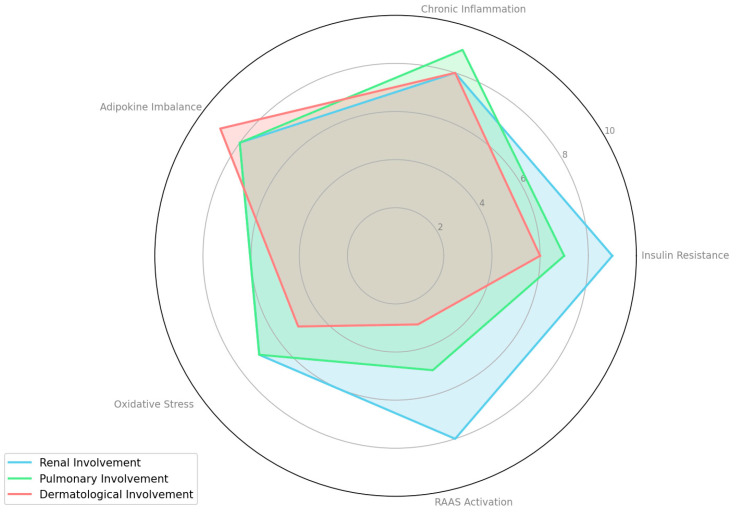
Comparative contribution of pathophysiological mechanisms to extrahepatic complications in MAFLD/NAFLD.

Overall, the table highlights the complex, multisystem nature of NAFLD/MAFLD. Insulin resistance, chronic inflammation, adipokine imbalances, oxidative stress, and RAAS activation each emerge as key pathophysiological drivers affecting multiple organs beyond the liver. Renal involvement is consistently underscored by mechanisms such as RAAS activation, fibrogenesis, and systemic inflammation. Pulmonary manifestations often center on hypoxia, endothelial dysfunction, and obesity-related stress, while dermatological issues are typically linked to the inflammatory and metabolic environment created by NAFLD/MAFLD. These relative contributions are also visualized in Figure 2, which presents a radar chart comparing the systemic impact of each mechanism across renal, pulmonary, and dermatological dimensions.

## 4. Diagnosis of MAFLD and Staging Criteria

The diagnosis of MAFLD represents a paradigm shift from the traditional NAFLD approach. Unlike NAFLD—which was defined by excluding other causes, such as significant alcohol consumption or viral hepatitis—MAFLD is diagnosed positively based on the presence of hepatic steatosis (as evidenced by imaging, histology, or biomarkers) in conjunction with metabolic dysfunction. Specifically, the diagnosis requires evidence of liver steatosis along with at least one of the following: overweight or obesity (BMI ≥ 25 kg/m²), type 2 diabetes mellitus, or the presence of at least two metabolic risk abnormalities (such as an elevated blood pressure >130/85 mmHg or being on antihypertensive therapy; high triglyceride levels >150 mg/dL; low HDL cholesterol (<40 mg/dL for men and <50 mg/dL for women); prediabetes, as defined by an HbA1c of 5.7–6.4% or fasting glucose of 100–125 mg/dL; increased HOMA-IR; or a high-sensitivity C-reactive protein concentration >2 mg/L) [28,29]. This redefinition, proposed by an international panel in 2020 and later endorsed by consensus documents [30,31], also permits the inclusion of patients with hepatic steatosis who consume moderate amounts of alcohol or have other concomitant liver conditions previously excluded under the NAFLD framework.

Following diagnosis, staging of MAFLD is critical to assess the risk of progression to more severe complications, such as cirrhosis or hepatocellular carcinoma (HCC). Staging is typically based on histological findings and can be broadly categorized as follows:

- *Simple Steatosis:* Characterized by fat accumulation in hepatocytes without significant inflammation or cellular injury, generally associated with a favorable prognosis.
- *Non-Alcoholic Steatohepatitis (NASH):* Marked by steatosis, lobular inflammation, and hepatocellular ballooning, often accompanied by early signs of fibrosis [32].
- *Hepatic Fibrosis:* Graded from F1 to F4, with F4 indicating cirrhosis. The extent of fibrosis is a key predictor of liver-related mortality [33].
- *Hepatocellular Carcinoma (HCC):* Can develop even in the absence of advanced fibrosis, particularly in the context of advanced NASH [34].

In clinical practice, various non-invasive methods, such as transient elastography (FibroScan), magnetic resonance elastography, and scoring systems (e.g., FIB-4, NAFLD Fibrosis Score), are routinely employed to estimate the degree of fibrosis.

## 5. Nephrological Manifestations of MAFLD/NAFLD

Patients with MAFLD/NAFLD have a higher prevalence of chronic kidney disease (CKD) than those without fatty liver disease, and the risk is even greater among those with advanced liver pathology (NASH or fibrosis) [5]. Insulin resistance (IR) contributes to glomerular hyperfiltration and damages the renal microvasculature. Elevated RAAS activity causes glomerular hypertension, promoting albuminuria and CKD progression [17]. Elevated inflammatory mediators accelerate nephron loss and renal fibrosis [4].

Early detection of CKD (through eGFR and urinary albumin measurements) is crucial. Rigorous management of metabolic risk factors (blood pressure, glycemic control, and lipid levels) can mitigate the progression of renal disease. Glomerular hyperfiltration and inflammation damage the glomerular basement membrane, leading to albuminuria, which can be an early sign of renal dysfunction [5]. Microalbuminuria or proteinuria is associated with a faster decline in renal function and heightened cardiovascular risk. Periodic screening for albuminuria/proteinuria in patients with MAFLD/NAFLD is essential.

Many individuals with MAFLD/NAFLD also have type 2 diabetes mellitus (T2DM) [4]. The overlapped pathophysiological processes (hyperglycemia, insulin resistance, oxidative stress) exacerbate kidney damage. Sodium–glucose cotransporter 2 (SGLT2) inhibitors and GLP-1 receptor agonists may benefit both glycemic control and renal protection in patients with T2DM and MAFLD/NAFLD [35,36].

According to a comprehensive meta-analysis that included approximately 64,000 participants from 20 cross-sectional and 13 longitudinal investigations, NAFLD was associated with a roughly twofold increase in the incidence (HR: 1.79; 95% CI: 1.65 to 1.95) and prevalence (OR: 2.12; 95% CI: 1.69 to 2.66) of CKD [37]. The majority of the time, noninvasive techniques (such as ultrasonography and scoring systems for fibrosis evaluation) were used to evaluate NAFLD; liver biopsies were used in a small number of instances.

According to certain studies, the histological severity of NASH, the progressive form of NAFLD, and the stage of hepatic fibrosis are associated with the extent of renal impairment [38]. According to the same meta-analysis, advanced fibrosis was associated with a higher incidence (HR: 3.29; 95% CI: 2.30 to 4.71) and prevalence (OR: 5.20; 95% CI, 3.14 to 8.61) of CKD than non-advanced fibrosis, and NASH was linked to a higher prevalence and incidence of CKD than simple hepatic steatosis [37]. Patients with a high risk of liver fibrosis had a 5.1-fold higher chance of developing chronic kidney disease (CKD) than low-risk patients (OR: 5.1; 95% CI: 1.13–23.28; *p* = 0.03), while subjects at intermediate risk had a 3-fold higher risk of liver fibrosis and a 3-fold higher risk of developing CKD compared to low-risk subjects (OR: 3.01; 95% CI: 0.87–10.32; *p* = 0.07) [39].

Jang et al. demonstrated in a recent longitudinal study that NAFLD is a separate risk factor related to the progression of CKD. Patients with advanced NAFLD (probably associated with a considerable or advanced hepatic fibrosis score) and those with reduced eGFR with or without proteinuria were at an increased risk of developing chronic kidney disease (CKD) [19].

Mantovani’s systematic review and meta-analysis of nine observational cohort studies, which included more than 96,500 middle-aged people of primarily Asian ethnicity during a median follow-up time of 5.2 years, revealed similar findings. The authors demonstrated that the risk of CKD in patients with NAFLD remained significant even after controlling for age, sex, obesity, hypertension, smoking, type 2 diabetes, baseline eGFR, or medications [20]. They also found a 40% increase in the long-term risk of incident CKD (random-effect HR: 1.37; 95% CI: 1.20–1.53; I2 = 33.5%) correlated with the severity of liver fibrosis.

In addition to having different genetic, nutritional, and adipose tissue distributions, the majority of the cohort studies described in meta-analyses are from Asian nations, where sizable populations participate in routine health examination programs [7]. Previous research has identified a disparity between eastern and western populations in regard to the connection between NAFLD and CKD. A large prospective cohort study of 8329 Korean men without T2DM, hypertension, or CKD at baseline followed for 4 years revealed that patients with NAFLD had a significantly higher risk of developing CKD after correcting for the same risk factors [40,41]. In contrast, the National Health and Nutrition Examination Survey (NHANES) study of 11,469 adults in the US did not show an increased risk of CKD in patients with NAFLD diagnosed by ultrasound after correcting for the presence of metabolic syndrome [12].

The mechanism linking NAFLD with renal impairment is yet unknown and the relationship between NAFLD and CKD is not yet well characterized. Furthermore, the relationship between NAFLD and CKD is reciprocal, meaning that kidney disease itself later aids in the advancement of liver damage, just like in people with type 2 diabetes [42]. Numerous studies indicate that oxidative stress, poor renin–angiotensin system regulation, and changes in the gut microbiota are common pathogenetic pathways between NAFLD and CKD [43]. According to now-available information, NAFLD may not be a sign of CKD, but rather a factor in the onset and progression of CKD [7].

In clinical practice, all NAFLD patients should have their renal function evaluated and tracked, much like patients with liver cirrhosis. Even in the absence of other traditional risk factors, people with NAFLD should be tested for CKD, according to a major meta-analysis by Musso et al. [37]. This is particularly advisable if severe fibrosis and/or NASH are suspected [44].

To stop the progression of CKD, reduce complications, and increase survival, it is essential to identify renal impairment in patients with NAFLD as soon as possible, even if there are no standards or surveillance techniques for this condition [9,45]. Since aberrant albuminuria (ACR > 30 mg/g), overt proteinuria, urine sediment abnormalities, and eGFR < 60 mL/min/1.73 m^2^ are all linked to a high or very high risk of disease development, clinicians should detect CKD at stage ≤3 [7]. We believe that all new patients with NAFLD should have a renal function assessment and that all medications that can affect kidney function in patients with NAFLD must be evaluated to allow for drug-dosage adjustments [46]. Armstrong et al. suggested that annual screening for CKD in patients with NAFLD by eGFR and microalbuminuria could detect early renal impairment in patients with NAFLD [27].

However, it is recommended that, to preserve the kidneys, nephrotoxic drugs should be used with care and prudence, and that NAFLD patients should be routinely evaluated for their renal function due to the increased risk of CKD in this group. NAFLD screening in CKD patients is not commonly performed, and there are no particular guidelines for NAFLD screening in CKD patients, despite research suggesting that NAFLD may accelerate the course of CKD. To ascertain the strategy and clinical management of NAFLD in individuals with chronic kidney disease, more research is necessary.

In addition, evidence highlights that patients with MAFLD exhibit a significantly increased prevalence of chronic kidney disease (CKD) compared to the general population. For instance, the NHANES (2017–2020) analysis reported a CKD prevalence of 24.7% among patients with MAFLD, compared to 8.3% in those with MASLD but without MAFLD criteria, with the relative risk of CKD being 4.73 times higher in the MAFLD group [47,48]. A Chinese study with over 8000 participants demonstrated a CKD prevalence of 8.9% in MAFLD patients versus 5.4% in the control group [49]. Moreover, a 10-year Japanese cohort study confirmed that MAFLD is an independent risk factor for CKD, with a hazard ratio of 1.12 [49]. Additionally, a recent review indicates that approximately 50% of CKD patients also have MAFLD, independent of diabetes or hypertension [50,51], while the prevalence of CKD in the general population remains between 0 and 71% [52]. These findings robustly support the concept that MAFLD is a multisystemic condition with significant renal impacts.

## 6. Pulmonary Manifestations of MAFLD/NAFLD

MAFLD/NAFLD is often associated with obesity, a key risk factor for obstructive sleep apnea (OSA) [1]. Evidence suggests a bidirectional relationship—OSA may exacerbate fatty liver disease and NAFLD may worsen OSA [35]. Recurrent nocturnal hypoxia and intermittent hypercapnia provoke systemic inflammation, amplifying insulin resistance. Altered adipokine secretion (TNF-α, IL-6) can further damage hepatic tissue. Patients with NAFLD should be tested for OSA symptoms (e.g., snoring, daytime somnolence). Continuous positive airway pressure (CPAP) therapy in OSA can improve liver enzymes and reduce systemic inflammation [35].

Obesity, common in MAFLD/NAFLD, increases the risk and severity of asthma [4]. Chronic inflammation shared between metabolic and respiratory disorders worsens lung outcomes. Emerging data suggest a link between NAFLD and reduced lung function in COPD, although large-scale evidence is still evolving. Systemic inflammation and dysregulated adipokines (e.g., leptin, adiponectin) contribute to airway hyperreactivity and remodeling [4]. Oxidative stress can exacerbate bronchial inflammation, particularly in patients with COPD overlapping and metabolic syndrome. Clinicians should evaluate respiratory symptoms in overweight or obese patients with MAFLD/NAFLD. Weight reduction can benefit both liver disease and pulmonary function.

Chronic inflammation and the increased cardiac workload from obesity can contribute to pulmonary artery hypertension (PAH). Although more commonly associated with cirrhosis and portal hypertension, mild or early elevations in pulmonary arterial pressure have been observed in metabolic syndrome. Some reports describe interstitial lung changes and reduced diffusion capacity in patients with NAFLD, but robust data is lacking [4]. Further research is needed to clarify these connections.

Patients with MetS and NAFLD who are obese experience upper airway collapse and airway edema, resulting in chronic intermittent hypoxia. This exacerbates insulin resistance and increases the incidence of NAFLD [53,54]. According to a number of studies, OSA causes liver damage and exacerbates NAFLD [55,56]. In both adults and children, there is a correlation between OSA and NAFLD [56,57]. A meta-analysis revealed that, regardless of age, sex, or body mass index, people with OSA had a greater chance of developing NAFLD, NASH, and advanced fibrosis [35]. Another meta-analysis found that OSA increased the risk of both advanced disease and NAFLD by two to three times. Even after adjusting for conventional risk factors, such as the patient’s age, sex, level of obesity, and body mass index, this risk persisted [35]. Screening obese patients with NAFLD for potential OSA, and screening those with existing OSA for underlying NAFLD is advised in light of these findings. Due to elevated leptin levels and the widespread proinflammatory state present in NAFLD, hyperresponsive airways with asthma are another pulmonary problem in these patients [58].

The usefulness and cost-effectiveness of screening all patients with NAFLD for OSA needs to be further investigated. Given the linked improvement in OSA severity and decreased upper airway collapsibility, behavioral changes to support weight loss are generally crucial and should be highly advised for patients with NAFLD and OSA [59,60].

Chronic intermittent hypoxia (CIH) is a term used to describe the recurrent episodes of upper airway blockage that occur during sleep in obstructive sleep apnea (OSA), a disorder associated with obesity [61]. Because CIH causes oxidative stress, it increases insulin resistance, endothelial dysfunction, vascular and systemic inflammation, and cardiovascular morbidity and mortality [62].

In obese adults (BMI > 37.8 kg/m^2^), Minville et al. [30] showed a dose–response association between the severity of nocturnal CIH and liver injury (as determined by non-invasive blood tests: SteatoTest, NashTest, and FibroTest). Lean OSA individuals did not reproduce this result. Furthermore, the authors showed that severe liver steatosis and borderline or possible NASH were associated with an increased risk of CVD in the context of OSA, as evidenced by higher blood pressure and more severe endothelial dysfunction [63]. According to these results, nocturnal hypoxemia must be accompanied by or precede preexisting obesity to advance from isolated liver steatosis to a more progressive phenotype of NASH. Additionally, endothelial dysfunction and subsequent cardiovascular disease may be caused by obesity in OSA patients. A two-step procedure is one suggested way to screen people with NAFLD for OSA: (1) give a questionnaire on the Epworth Sleepiness Scale, and (2) conduct nighttime surveillance on individuals who are highly at risk [35]. However, OSA screening questionnaires have not been explicitly validated in NAFLD patients and have relatively low sensitivity and specificity [35]. In addition, monitoring the progression of the condition and looking at potential underlying NAFLD should be considered in obese patients with OSA.

A major mediator of OSA, intermittent hypoxia (IH) causes a number of changes in physiological homeostasis [64]. Through carotid body chemoreceptors, it triggers the sympathetic nervous system, which causes adipose tissue to break down into fat (lipolysis). Triglycerides are made up of free fatty acids (FFAs) and are then transferred to various organs for use as fuel. However, FFAs can cause organ inflammation and steatosis, as in the case of NAFLD. Simultaneously, IH triggers the activation of hypoxia-inducible factors 1 and 2 (HIF-1 and HIF-2, respectively), which results in increased hepatic fat production, reduced lipid metabolism, increased inflammation, and fibrosis in the liver. Vgontzas et al. also mentioned the generation of inflammatory cytokines, which caused oxidative stress in patients with chronic sleep apnea and could be a potential mechanism influencing the pathophysiology of non-alcoholic fatty liver disease. In addition to increasing IR in NAFLD patients, this process may quicken the evolution of liver fibrosis, which can lead to cirrhosis, steatohepatitis, and other problems [35,44].

Although pulmonary complications have been less extensively explored compared to cardiometabolic issues, pulmonary involvement is increasingly recognized in MAFLD. Patients with MAFLD exhibit restrictive ventilatory dysfunction and significant systemic inflammation associated with obesity and insulin resistance [65]. Moreover, respiratory disorders, such as obstructive sleep apnea (OSA) and chronic obstructive pulmonary disease (COPD), are significantly more prevalent among patients with MAFLD. For instance, the prevalence of MAFLD reaches 78% in patients with severe OSA, compared to 58% in those without OSA [66]. Additionally, the overlap of OSA and COPD has been reported in up to 65.9% of patients with chronic respiratory disease, a marked contrast to the 6–13% prevalence observed in the general population [67]. At a population level, the prevalence of chronic pulmonary diseases (especially COPD) is approximately 4–20%, depending on risk factors such as smoking and pollution [68]. These differences support the integration of respiratory function evaluations into the screening algorithm for patients with MAFLD.

## 7. Skin Manifestations of MAFLD/NAFLD

MetS and NAFLD are more common in patients with psoriasis, a chronic autoimmune inflammatory skin disease. The degree of psoriasis is directly correlated with this connection [69,70]. It is uncertain whether NAFLD patients have a higher incidence of psoriasis. It is still unclear if this association is actually caused by psoriasis or by the medications used to treat it, particularly methotrexate. Acanthosis nigricans, keratosis, and hirsutism are additional dermatological conditions in these patients brought on by underlying insulin resistance [60]. In addition, obesity in these patients leads to malfunctioning sweat and sebaceous gland activity, as well as decreased skin barrier function. Additionally, obesity has a mechanical effect that increases the frequency of varicose veins, which can lead to cellulitis, fasciitis, lymphedema, and varicose ulcers [60].

Tumor necrosis factor (TNF)-α and other inflammatory cytokines are believed to be involved in the relationship between NAFLD and psoriasis, although the precise mechanism underlying this correlation is still unknown [71]. According to a case series, 89 patients with psoriasis, metabolic syndrome, and non-alcoholic fatty liver disease (NAFLD) who were treated with etanercept (a TNF-α inhibitor), as opposed to psoralen–ultraviolet A, showed a significant decrease in their fasting insulin, aspartate aminotransferase/alanine transaminase ratio, homeostasis model assessment index, and C-reactive protein levels [72]. TNF-α inhibitors may help patients with NAFLD with their psoriasis, but more extensive cohort studies are needed to validate this conclusion.

In addition, cutaneous manifestations, particularly psoriasis, are increasingly reported in association with MAFLD, reflecting shared systemic inflammation and metabolic dysfunction. An analysis by Kaya and Yılmaz indicates that the prevalence of psoriasis in patients with MAFLD ranges between 3 and 142%, compared to only 2–3% in the general population [73]. Furthermore, the severity of skin involvement correlates with the degree of hepatic inflammation and fibrosis, and patients with psoriasis are at a significantly increased risk of developing MAFLD [74]. These data support the inclusion of routine dermatological evaluations in the management of patients with MAFLD, especially in the presence of chronic inflammatory skin diseases.

**Figure 3:**
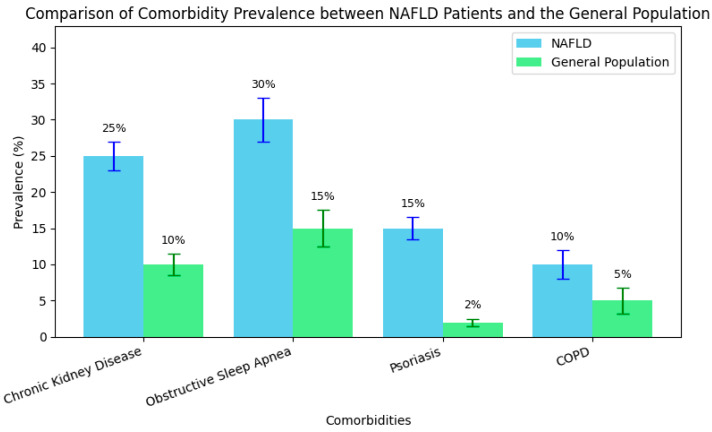
Comparative prevalence of key comorbidities in NAFLD patients vs. the general population.

These epidemiological differences in comorbidity prevalence between NAFLD patients and the general population are further illustrated in Figure 3, which highlights the disproportionately high rates of renal, pulmonary, and dermatological conditions—especially psoriasis—among individuals with NAFLD.

## 8. Clinical Management Strategies

### 8.1. Screening and Early Detection

Annual screening for albuminuria and eGFR is recommended, particularly in patients with T2DM or hypertension [36]. We recommend evaluating for OSA, especially in obese patients, and considering spirometry for patients with respiratory symptoms or risk factors for asthma/COPD.

### 8.2. Lifestyle Modifications

#### 8.2.1. Weight Loss

Caloric restriction and increased physical activity reduce liver fat, improve insulin sensitivity, and benefit both kidney and lung health [4]. A 5–10% weight reduction can significantly improve liver histology and metabolic parameters. According to the American Association for the Study of Liver Diseases’ practice guidelines, NAFLD may be improved by losing at least 3% to 5% of body weight; however, the degree of liver necroinflammation may need to be improved by losing up to 10% of body weight [75]. In contrast, a consistent 5–7% weight loss may be enough to reduce the risk of type 2 diabetes [76]. Even when weight loss was generally moderate, recent intervention trials have demonstrated a considerable reduction in the risk of type 2 diabetes (T2DM) with weight reduction (from 42% to 67%) compared to control groups [77,78]. Improvements in peripheral insulin sensitivity, insulin secretory responses, and cellular insulin signal transduction are all associated with weight loss [78]. It appears that patients with NAFLD who have patatin-like phospholipase domain-containing protein 3 (PNPLA3) are more susceptible to the positive effects of lifestyle changes on liver steatosis [49]. According to two European investigations, people with homozygous GG experience an even greater reduction in intrahepatic triglyceride concentration [79] and liver enzymes [80] when they lose weight than subjects with homozygous CC. Although the etiology of this discovery is currently unclear, it may be explained by variations in insulin sensitivity between the two genotypes and the impact of abdominal obesity, which may alter the way the PNPLA3 G allele causes liver damage [49].

#### 8.2.2. Nutritional Interventions

Balanced diets low in refined carbohydrates and saturated fats (e.g., the Mediterranean diet) can improve NAFLD/MAFLD [1]. A higher fiber intake helps with glycemic control and reduces inflammation.

Several dietary approaches may be beneficial for CVD, metabolic syndrome, and NAFLD. Although there is currently no consensus regarding advice for dietary therapy to treat NAFLD, metabolic syndrome, and CVD, reducing total calorie intake is an essential part of lifestyle adjustment. Ethnicity, sex, BMI, and comorbidities all affect daily calorie intake. It is often advised to estimate each person’s energy needs and prescribe an energy deficit of 500 kcal per day, or 30% of baseline, in order to achieve the best caloric reduction [81]. To treat NAFLD, concomitant metabolic syndrome, and/or cardiovascular disease, a modification in the diet content may be crucial, in addition to lowering overall calorie intake. NAFLD subjects typically eat less omega-3 fatty acid–rich seafood and more meat and fizzy drinks [82]. According to recent systematic studies, dietary fat (such as total and saturated fat) and carbohydrate (such as simple and high glycemic carbohydrate) restriction can lower liver enzymes and/or lessen the grade of steatosis in NAFLD patients [83,84]. Restricting fructose intake may be advantageous insofar as it is associated with advanced histopathology and non-alcoholic fatty liver disease (NAFLD) [85].

#### 8.2.3. Physical Activity

By lowering visceral fat, increased physical activity is believed to help NAFLD and its concomitant diseases, such as metabolic syndrome and CVD. According to a number of studies, a decrease in visceral fat preceded a decrease in liver fat. Therefore, even though there are contradictory findings, the association between hepatic fat content and physical activity vanishes when intra-abdominal obesity is taken into consideration [86,87]. Regardless of visceral obesity and insulin resistance, a large cross-sectional investigation found an inverse relationship between the prevalence of NAFLD and total and leisure-time physical activity [88]. Our group recently conducted a prospective cohort study that showed that the physical activity level at baseline was associated with a decreased risk of incident NAFLD in a 4-year follow-up [89]. Furthermore, regardless of visceral adiposity and insulin resistance, prolonged or increased physical activity prevented the incidence of non-alcoholic fatty liver disease [89]. In conclusion, regardless of visceral obesity or insulin resistance, increased physical activity is a crucial part of lifestyle change in individuals with NAFLD and concomitant extrahepatic symptoms. Regarding the best workout routine, including its duration and the kind of activities, there is disagreement.

### 8.3. Pharmacotherapy

#### 8.3.1. Metformin

A first-line therapy for T2DM, metformin may offer modest benefits for hepatic steatosis and stabilizing eGFR [4]. SGLT2 inhibitors (e.g., empagliflozin, dapagliflozin) are effective for T2DM and CKD; ongoing trials are examining their direct effects on NAFLD [16]. GLP-1 receptor agonists (e.g., liraglutide, semaglutide) help patients to achieve substantial weight loss and can improve liver steatosis; they also show renal protective properties [36].

#### 8.3.2. Anti-Hypertensive Agents (ACE Inhibitors, ARBs)

ACE inhibitors may reduce RAAS-mediated renal damage and slow NAFLD progression through anti-inflammatory effects [17].

### 8.4. Multidisciplinary Approach

Coordination among hepatologists, nephrologists, pulmonologists, dermatologists, nutritionists, endocrinologists, and primary care providers is essential [4]. Integrated care systems can optimize risk-factor management, improve adherence, and enhance outcomes. We emphasize the importance of lifestyle changes, adherence to medications, and regular screening, and recommend that clinicians encourage smoking cessation to reduce cardiovascular and lung risks.

## 9. Future Directions

Large-scale, long-term studies are needed to clarify the precise mechanisms linking NAFLD/MAFLD with kidney, lung, and skin dysfunction [1,2,3]. Novel biomarkers may allow for earlier detection and more targeted interventions. Genetic and epigenetic factors influencing susceptibility to renal and pulmonary complications in MAFLD/NAFLD are under investigation. Tailoring treatments based on genetic profiles and advanced imaging/lab data could optimize outcomes. New anti-fibrotic and metabolic agents (e.g., FXR agonists, dual incretin agonists) are in clinical trials and may provide organ-protective benefits beyond the liver [4].

## 10. Conclusions

MAFLD/NAFLD is increasingly recognized as a multisystemic disorder with significant implications for renal, pulmonary and skin health. Chronic kidney disease, albuminuria, obstructive sleep apnea, and psoriasis are among the most commonly observed extrahepatic manifestations, while more research is needed to clarify the relationship with asthma, COPD, and pulmonary hypertension. A proactive and multifaceted approach—including screening, lifestyle interventions, pharmacotherapy tailored to metabolic and organ-specific needs, and close interdisciplinary collaboration—remains the cornerstone of care. Ongoing research aiming to elucidate pathophysiological mechanisms and develop novel treatments holds promise for attenuating the burden of MAFLD/NAFLD on the kidneys, lungs, and skin. The cornerstone of treatment for many extrahepatic manifestations linked to NAFLD is lifestyle modification, including appropriate weight loss, physical activity, and dietary modifications, although the current guidelines do not offer guidance on the management of these manifestations.

To diagnose extrahepatic manifestations of NAFLD, clinical practice can take into account suggested screening methods for common comorbidities in NAFLD, such as obtaining fasting blood glucose, hemoglobin A1c, lipid profiles, estimated glomerular filtration rate, urine microalbumin, and the albumin–creatinine ratio. The impact of pharmacologic treatment on extrahepatic symptoms of non-alcoholic fatty liver disease requires more research.

## Figures and Tables

**Table 1:** Scoring the multisystemic impact of key pathophysiological mechanisms in MAFLD/NAFLD based on the literature review.

Study	Main Mechanism	Renal Involvement (Score)	Pulmonary Involvement (Score)	Dermatological Involvement (Score)	Observations
Musso et al. [14]	Insulin Resistance	9	7	6	Fully confirms the role of both hepatic and peripheral insulin resistance (IR) in the pathogenesis of NAFLD/MAFLD, highlighting its direct contribution to systemic inflammation, endothelial dysfunction, and kidney damage. IR is also implicated in hypoxia- and oxidative stress–related lung changes and skin manifestations (e.g., psoriasis, acanthosis nigricans).
Byrne and Targher [4]	Chronic Inflammation	8	9	8	Emphasizes NAFLD/MAFLD as a multisystem disease. Shows that NAFLD is an independent risk factor for chronic kidney disease (CKD). Systemic inflammation and endothelial dysfunction are strongly linked to pulmonary issues and are also associated with inflammatory skin conditions, such as psoriasis.
Khan et al. [28]	Adipokine Imbalance	8	8	9	Focuses on how alterations in adipokine levels (reduced adiponectin, increased leptin) contribute to NAFLD progression and systemic insulin resistance. Leptin promotes fibrogenesis and oxidative stress in the kidneys, while widespread inflammatory effects influence pulmonary function and are closely linked to dermatological manifestations (e.g., psoriasis).
Kuchay et al. [29]	Oxidative Stress	7	7	5	Examines oxidative stress as a key driver in MAFLD, contributing to both hepatic and extrahepatic tissue damage. While there is moderate emphasis on renal and pulmonary impact via systemic inflammation and obesity-related factors, skin involvement is less thoroughly detailed, hence the lower dermatological score.
Minville et al. [30]	RAAS Activation	8	5	3	Investigates NAFLD in patients with obstructive sleep apnea (OSA), focusing on nocturnal hypoxia and endothelial dysfunction. RAAS activation is implicated indirectly through hypoxia-induced stress and vascular effects, which can influence renal function. Pulmonary involvement is mentioned but not deeply explored, and dermatological involvement is minimally referenced.

## Data Availability

No new data were created or analyzed in this study.
